# Association between Excessive Dietary Branched-Chain Amino Acids Intake and Hypertension Risk in Chinese Population

**DOI:** 10.3390/nu14132582

**Published:** 2022-06-22

**Authors:** Yuyan Liu, Chengwen Zhang, Yuan Zhang, Xuheng Jiang, Yuanhong Liang, Huan Wang, Yongfang Li, Guifan Sun

**Affiliations:** 1 Department of Clinical Epidemiology, The Fourth Affiliated Hospital of China Medical University, Shenyang 110031, China; yyliu@cmu.edu.cn; 2 Research Center of Environmental and Non-Communicable Disease, School of Public Health, China Medical University, Shenyang 110000, China; ailsa-zhang@163.com (C.Z.); rozy120189@163.com (Y.Z.); joslynj@126.com (X.J.); lyhnsmc@163.com (Y.L.); whjoyce2021@163.com (H.W.); gfsuncmu@163.com (G.S.)

**Keywords:** branched-chain amino acids, isoleucine, leucine, valine, hypertension

## Abstract

The dietary intake of branched-chain amino acids (BCAAs) has been reported to be associated with both elevated blood pressure (BP) and hypertension risk, while published findings were inconsistent, and the causality has never been well disclosed. We performed this prospective study aiming to find out the relationship between dietary BCAAs intake and hypertension risk in the Chinese population. A total of 8491 participants (40,285 person-years) were selected. The levels of dietary BCAAs intake were estimated using the 24-h Food Frequency Questionnaire. Associations of both BP values and hypertension risk with per standard deviation increase of BCAAs were estimated using linear and COX regression analysis, respectively. The hazard ratios and 95% confidence interval were given. Restricted cubic spline analysis (RCS) was used to estimate the nonlinearity. Both systolic and diastolic BP values at the end points of follow-up were positively associated with dietary BCAAs intake. Positive associations between BCAAs intake and hypertension risk were shown in both men and women. By performing a RCS analysis, the nonlinear relationship between BCAAs intake and hypertension was shown. As the intake levels of Ile, Leu, and Val, respectively, exceeded 2.49 g/day, 4.91 g/day, and 2.88 g/day in men (2.16 g/day, 3.84 g/day, and 2.56 g/day in women), the hypertension risk increased. Our findings could provide some concrete evidence in the primary prevention of hypertension based on dietary interventions.

## 1. Introduction

Isoleucine (Ile), leucine (Leu), and valine (Val), are essential amino acids with the common special structure of nonlinear aliphatic side chains, and named as branched-chain amino acids (BCAAs) [1,2]. As critical metabolic intermediates, BCAAs play an important role in regulating glucose intake and protein synthesis [1,3,4]. However, adverse effects of BCAAs have been frequently reported in recent years. The potential underlying mechanisms might include the extra skeletal muscle fatty acid uptake caused by 3-hydroxy-isobutyrate (3-HIB) decomposed from BCAAs, and the impairment of the liver tricarboxylic acid (TCA) cycle caused by branched-chain α-keto acids (BCKAs), which is another catabolic intermediate of BCAAs [5,6,7]. All of these adverse catabolites that are derived from the excessive accumulation of BCAAs could trigger and aggravate the development of chronic inflammation and insulin resistance (IR), and thereafter link BCAAs to various cardiovascular diseases (CVDs) [8,9]. Population-based epidemiological studies have disclosed that the level of BCAAs estimated using both food frequency questionnaire (FFQ) and blood samples was positively associated with several CVD-relevant biomarkers, including triglycerides (TG), homeostatic model assessment (HOMA-IR), and carotid intima-media thickness (IMT) [10,11,12,13].

Elevated blood pressure (BP) and hypertension are major risk factors of CVDs, and generate huge health and economic burdens [14,15,16]. The clarification of more modifiable risk factors for hypertension has received considerable interest. Despite that associations between BCAAs and hypertension have been estimated in some population-based studies, such relationship still remained inconclusive, and the causality was uncertain due to the lack of relevant prospective studies [17,18,19,20,21,22]. In addition, given that the beneficial roles of BCAAs as essential amino acids could not be neglected, we hypothesized that the dietary BCAAs intake probably did not increase hypertension risk unless the level of dietary intake was beyond a proper range. However, the nonlinear relationship between the intake of BCAAs and hypertension risk has never been disclosed in previous studies. Moreover, since the Chinese population has been suffering from an increasing incidence of hypertension and huge changes in dietary habits to western patterns during the past few decades, it is critical to provide some favorable guidance on the dietary intake of nutrients for Chinese people [23,24]. However, no cohort study has so far been performed in Chinese people to reveal the relationship between dietary BCAAs intake and hypertension risk. 

Therefore, we performed this large-scale prospective study to explore the association between dietary BCAAs intake and hypertension risk among the Chinese population. We aimed to fill the gap in the field of population-based studies concerning BCAAs intake and hypertension risk, and to clarify whether a nonlinear relationship existed.

## 2. Materials and Methods

### 2.1. Study Design and Populations

The China Health and Nutrition Survey (CHNS) is a nationwide cohort project aiming to examine nutritional and health behaviors, as well as relevant outcomes among general Chinese residents [25]. From 1989 to 2011, nine waves of follow-up investigations (i.e., 1989, 1991, 1993, 1997, 2000, 2004, 2006, 2009, and 2011) were completed in 15 provinces and municipal cities [26]. We conducted this current longitudinal study based on the data from four waves of the investigations from 2004 to 2011. Briefly, 23,425 participants (51,865 person-waves) in total were investigated from 2004 to 2011, and 9637 were investigated in only one wave, i.e., about 41.1% were lost to follow-up. Therefore, 13,788 participants (42,228 person-waves) investigated in two or more waves were included (Figure S1, Supplementary Materials). We further excluded those who were not eligible for the following analyses based on our exclusion criteria, including those aged younger than 18 years old, pregnant women, those with a history of myocardial infarction, being diagnosed as hypertension at baseline, and any participant with missing data of dietary BCAAs intake. As a result, 8491 participants (24,945 person-waves, i.e., 40,285 person-years) remained for the subsequent analysis. The CHNS was approved by the Institutional Review Boards at the University of North Carolina at Chapel Hill (#07–1963), the National Institute for Nutrition and Health, and the Chinese Center for Disease Control and Prevention (#201524). All of the participants provided written informed consent [27]. 

### 2.2. Information of Dietary BCAAs Intake

In each investigating wave of the CHNS, dietary information was collected via face-to-face interviews by trained nutritionists, based on FFQ of 24-h dietary recalls during the past 3 days which were randomly allocated from Monday to Sunday in the past 1 week. The FFQ included both categories and amounts of food and meals at both individual and household levels. The places of consumption were collected individually, and the consumptions of cooking oil and condiments were collected per household. The accuracy of the 24-h dietary recall designed to assess nutritional intake has been validated [28]. Thereafter, based on the total intake of each individual food consumed across the duration of 3 days, the 3-day amount of the dietary intake of Ile, Leu, and Val, as well as energy, carbohydrate, fat, protein, salt and sodium were then estimated for each of the participants using the China Food Consumption Table 2002; Table 2004 (FCT 2002 and 2004), in which the levels of each single dietary ingredient of various foods were shown in detail. Considering that the dietary food codes used in the waves of 1989, 1991, 1993, 1997, and 2000 could not be matched with those in the FCTs, we then only included the data from 2004 in the analysis. To accurately reflect the daily intake of BCAAs, we firstly summed up all of the intake values of each individual BCAA from the first wave of investigation to the end (diagnosed as hypertension or not) for each participant, and then divided these values by the total number of days that people consumed these nutrients during the follow-up (number of waves multiplied by three) to calculate the amount of dietary intake per day.

### 2.3. BP Measurements and the Definition of Hypertension

BP was measured using a standard mercury-column sphygmomanometer with an appropriate adult upper arm cuff size after 5 min of rest in the sitting position, based on the standardized procedural guideline. Systolic BP (SBP) and diastolic BP (DBP) were determined by the first and the fifth Korotkoff sounds. The BP on both right and left arms was measured first, and the arm with higher blood pressure would be selected to obtain three consecutive measurements with a time interval of at least 1 min. Then, the average on the selected arm would be recorded as the final BP. The participants were also asked not to do any of the following behaviors: drinking alcohol, tea or coffee; smoking; or taking any exercise for at least 30 min before measuring the BP [29,30]. In this study, we defined hypertension as SBP ≥ 140 mmHg, or DBP ≥ 90 mmHg, or use of antihypertensive medication.

### 2.4. Measurements of Confounders

All of the participants were asked to undergo anthropometric measurements while wearing light clothes without shoes. The body weight was measured using a vertical weight scale. The height and waist circumference (WC) were measured using a metric scale. The BMI was then calculated as weight (kg) divided by the square of the height (m). According to the currently used cutoff point of BMI in defining the overweight and obesity in the Chinese population [31], we defined the overweight or obesity as BMI ≥ 24 kg/m^2^. The central obesity was defined as WC ≥ 85 cm in men or ≥80 cm in women [32]. In this study, type 2 diabetes mellitus (T2DM) was identified according to self-reports of a history of T2DM diagnosis; or receiving any treatment for T2DM, including a special diet, weight control, oral medication, insulin injections, Chinese traditional medicine, or home remedies [33,34]. 

In addition, self-administered questionnaires were also used to obtain information on demographic characteristics, including urban residence, nationality, education level, and status of smoking and alcohol drinking. Trained staff members confirmed the reported information with each participant. In our study, physical activity level was defined as the combination of both occupational and home activities, as previously reported. The total metabolic equivalents (METs) of physical activity multiplied by hours per week (METs-hours/week) were then calculated, according to the formulas [35]. Considering that the distribution of the values of METs-hours/week did not follow a bell-shape and strongly skewed in the test of normal distribution, we performed a log-transformation for this variable before the following analyses.

### 2.5. Statistical Analysis

All of the continuous variables were presented as mean and standard deviation (SD). The categorical variables were presented as percentages. The Student’s *t*-test and chi-square test were used to estimate the differences of continuous and categorical variables between men and women, respectively. 

The adjusted associations of both SBP and DBP at the end of the follow-up duration with per SD increase of each individual BCAA (Ile, Leu, and Val) were, respectively, estimated using multivariable linear regression analyses among the participants without any anti-hypertensive treatment. The relationships between changes of BP values (i.e., the differences of BP values between the baseline and end points of follow-up) and the intake of dietary BCAAs were also analyzed, using the same methods as above mentioned. Multivariable COX regression analyses were used to estimate the association between hypertension risk and per SD increase of dietary BCAAs intake, and the results were shown as hazard ratio (HR) and 95% confidence interval (95% CI). Before performing the COX regression analysis, the Kaplan–Meier curve of probability of non-hypertension was generated, and the diagnostic of proportional hazards assumption based on weighted Schoenfeld residuals was performed. Moreover, we further divided the participants into four subgroups based on the quartiles of Ile, Leu, and Val, respectively, and then performed both linear and COX regression analyses, using the subgroup of the lowest level of BCAAs intake (Q1) as the reference. In addition, the COX regression analysis was also estimated separately in each subgroup of BCAAs quartiles (from Q1 to Q4). The COX regression analyses were performed in three models: Model 1 (adjusted for age); Model 2 (adjusted for ethnicity, education, urban residents, T2DM, physical activity, smoking and alcohol drinking in addition to Model 1); and Model 3 (adjusted for intakes of energy, carbohydrate, fat, protein, and salt intake in addition to Model 2). We completed these analyses in both men and women, respectively, and the *p*-values of the interaction by gender were given. The interaction by gender was only estimated for the BCAAs, not for any other confounder (e.g., by running the SAS code “PROC PHREG; MODEL time*hypertension (0) = BCAAs gender BCAAs*gender age ethnicity education……protein salt; RUN;”).

To find out the non-linear relationship between BCAAs and hypertension, restricted cubic splines (RCS) analyses were performed. Three knots at the 10th, 50th, and 90th centiles of Ile, Leu, and Val were used and the median values were set as the reference. The RCS analyses were performed in men and women, respectively, and adjusted for consistent confounders with those in Model 3 above mentioned. In addition, stratified analyses of the associations between BCAAs and hypertension risk were also performed, based on the following categorical variables: age (<40, 40–65, and ≥65 years old); BMI (overweight/obesity or not); WC (central obesity or not); intakes of energy, fat, protein, carbohydrate and sodium (divided using medians, respectively); smoking status (yes/no); and alcohol drinking (yes/no). 

All of the above statistical analyses were completed using SAS 9.4 (SAS Institute, Inc., Cary, NC, USA) and R software (v.4.0.3). A *p*-Value < 0.05 was considered as statistically significant.

## 3. Results

### 3.1. General Characteristics

The characteristics of the 8491 participants (3995 men) are shown in Table 1 as mean ± SD and percentages. The averages of age were 44.4 and 44.8 years old, and follow-up durations were 4.68 and 4.81 years in men and women, respectively. In our specific participants, we found that the intakes of energy, carbohydrate, fat and protein were generally higher in men, so were the intakes of Ile, Leu, and Val. Neither salt nor sodium intake showed a difference between men and women. Both SBP and DBP were higher in men, with *p*-values < 0.001, so was the change of DBP during the follow-up. The hypertension incidence was 28.9% and 24.6% in men and women, respectively. 

### 3.2. Associations of BP Values with Dietary BCAAs Intake

The results of the multivariable linear regression analysis showed that both SBP and DBP at the end points of the follow-up significantly increased per SD increase in the dietary intakes of Ile, Leu, and Val (Table 2). In contrast with Leu and Val, the dietary intake of Ile was stronger associated with the increase of both SBP and DBP. Positive associations of changes of both SBP and DBP with BCAAs intake were found in both men and women, and the interaction by gender was only shown for changes of SBP. As per SD increase in BCAAs intake, the changes of SBP were larger in men (*p*-values for interaction by gender were 0.008, 0.020, and 0.012 for Ile, Leu, and Val, respectively). To further confirm the association between BCAAs intake and BP, we transferred the values of BCAAs intake into categorical variables using quartiles. The findings revealed that in the Q4 subgroup of BCAAs intake, both the BP values at the end point of follow-up and the changes during the follow-up period were significantly larger than those observed in Q1 (Tables S1 and S2, Supplementary Materials). 

### 3.3. Associations of Hypertension Risk with Dietary BCAAs Intake

As shown in Table S3 (Supplementary Materials), the probabilities of non-hypertension in men and women were 0.622 and 0.680, respectively, and the Kaplan–Meier curve is shown in Figure S2 (Supplementary Materials). The results from the diagnostics of proportional hazards’ assumption are shown in Figure S3 (Supplementary Materials). In both men and women, a zero slope was shown for each individual BCAA, indicating that the effect of the BCAAs on hypertension risk did not vary with time. 

In the multivariable COX regression analysis, positive associations between all three of the BCAAs and hypertension risk were found in both men and women, after the adjustment for age in Model 1 (*p*-values < 0.001), no interaction by gender was found (Table 3). By further adjusting for confounders in Model 2 and 3, consistent results were shown. Compared to the dietary intakes of Leu and Val, Ile was slightly stronger associated with hypertension. For example, in Model 3, the HRs for Ile, Leu, and Val in men were 1.24 (95% CI: 1.14, 1.35), 1.23 (95% CI: 1.13, 1.33), and 1.23 (95% CI: 1.13, 1.33), respectively. The HRs in women were 1.29 (95% CI: 1.19, 1.40), 1.27 (95% CI: 1.18, 1.38), and 1.28 (95% CI: 1.18, 1.38) for Ile, Leu, and Val, respectively.

**Figure 1:**
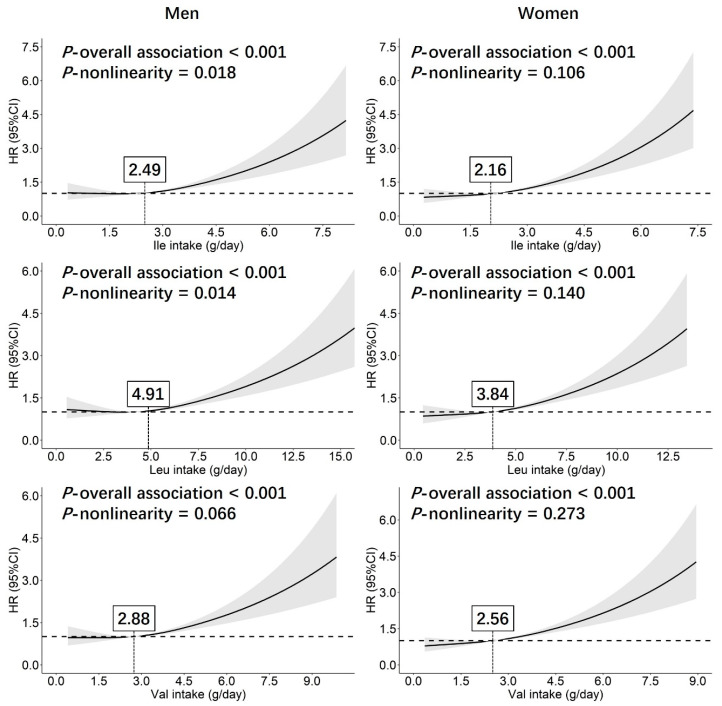
Nonlinear relationship between dietary BCAAs’ intake and hypertension risk. The estimate of nonlinearity was adjusted for age, ethnicity, education, urban residents, diagnosed T2DM, physical activity, smoking, alcohol drinking, intakes of energy, carbohydrate, fat, protein, and salt intake. Abbreviations: BCAA: branched-chain amino acid; Ile: isoleucine; Leu: leucine; Val: valine; HR: hazard ratio; 95% CI: 95% confidence interval.

By dividing the participants into subgroups using quartiles of Ile, Leu, and Val, the incidence of hypertension generally increased in the subgroups with higher BCAAs intake in both men and women, while significant differences were only shown between the subgroups of Q4 and Q1 in the COX regression analysis (Table S4, Supplementary Materials). The COX regression analysis using the continuous variables separately in different subgroups from Q1 to Q4 of BCAAs showed that for both men and women, all three of the BCAAs were positively associated with hypertension risk only in the subgroup of Q4 (Table 4). The results from the nonlinearity analysis performed using RCS models revealed that the nonlinear relationships between BCAAs intake and hypertension were shown in both men and women, while significant *p*-values for nonlinearity were only found in men (*p*-values for nonlinearity were 0.018, 0.014 and 0.066 for Ile, Leu, and Val, respectively). As the dietary intake levels of Ile, Leu, and Val, respectively, exceeded 2.49 g/day, 4.91 g/day, and 2.88 g/day in men (2.16 g/day, 3.84 g/day, and 2.56 g/day in women), the hypertension risk increased (Figure 1). 

**Figure 2:**
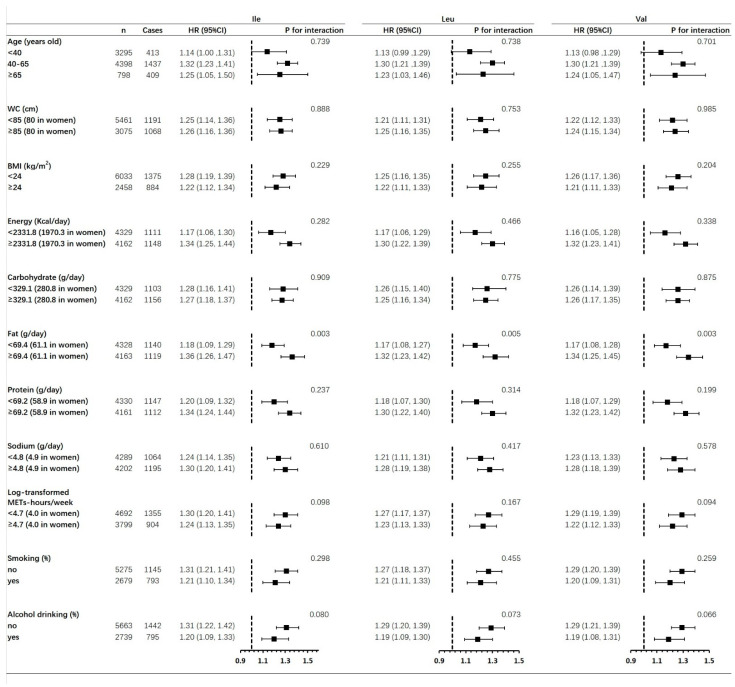
Stratified analysis of relationship between dietary BCAAs intake and hypertension risk based on confounders. HRs were estimated as per SD increase in BCAAs. Regression models were adjusted for age, gender, ethnicity, education, urban residents, diagnosed T2DM, physical activity, smoking, alcohol drinking, intakes of energy, carbohydrate, fat, protein, and salt intake. The interaction was only estimated for BCAAs, not for any other confounder. Abbreviations: BCAA: branched-chain amino acid; Ile: isoleucine; Leu: leucine; Val: valine; BMI: body mass index; WC: waist circumference; HR: hazard ratio; 95% CI: 95% confidence interval.

The stratified analysis using the relevant confounders showed that only an interaction by dietary fat intake existed, and stronger associations between BCAAs and hypertension showed in those with a higher fat intake (Figure 2). Although no significant interaction was found, larger HRs were still shown in those with higher intakes of energy, protein, and sodium. Moreover, stronger relationships were found in those with higher level of physical activities, without cigarette smoking, and without alcohol drinking, respectively.

## 4. Discussion

In summary, we found that both SBP and DBP at the end point of follow-up were positively associated with dietary BCAAs intake in men and women. Positive relationships between dietary BCAAs intake and hypertension incidence existed, while in further regression analysis separately in subgroups divided by quartiles of BCAAs, statistically significant HRs were only found in people with the highest level of BCAAs intake. The findings from the nonlinearity analysis showed that there was a nonlinear relationship between the BCAAs intake and hypertension risk.

Although associations of BCAAs with both elevated BP and hypertension have been reported, the findings were inconsistent. For example, there is a cross-sectional study performed with 361 Caucasian men showing that the plasma levels of BCAAs were positively associated with BP and hypertension [36]. Similar results were also reported in a case-control study of 5541 Japanese [19]. However, another Japanese study disclosed that, after adjusting for confounders, the relationship between plasma BCAAs and hypertension was abolished [37]. Hu et al. also reported that serum BCAAs were not associated with either SBP or DBP [38]. Considering that all of the above introduced studies were not of a longitudinal design, the causality between BCAAs and increased hypertension risk could not be found. Although a more recent prospective study of 4169 Netherland participants disclosed such causality, the generalizability of this finding still remains underdetermined, given the variances in genetics and dietary habits among different populations [22]. 

The current methods of estimating levels of BCAAs include both the investigation of dietary intake using FFQ, and the examination of plasma or serum levels. The examination based on blood samples could give a more accurate estimate, while higher requirements for both equipment and technologies, as well as the expensive cost limit its utility among general populations. Moreover, experimental studies have shown that the situation of IR might also in turn elevate the levels of BCAAs in blood [39]. As a result, the relationship between the circulating level of BCAAs and relevant metabolic disorders, including hypertension, could not be clearly disclosed in a cross-sectional design, which perhaps contributed to the inconsistency of published results. In contrast, FFQ investigation as an economical and easily operated method, could provide information about people’s dietary habits during a certain period in a relatively realistic way. It has been reported that 80% of circulating BCAAs were derived from dietary intake [5,40], ensuring that at least to some extent, FFQ investigations could reflect the internal levels of BCAAs, and correspondingly could be used to analyze the relationship between BCAAs and relevant metabolic disorders. For example, in a 3-year follow-up study in 4288 participants from Iran, the dietary intake of Val was found to be associated with increased hypertension risk [41]. In our study, we performed a prospective study with 8491 participants based on the CHNS cohort, and as we know, it was the largest sample size so far among the published studies for disclosing the causality between dietary BCAAs intake and hypertension risk. Moreover, we summed up the amounts of dietary BCAAs intake at different time points during the follow-up and then averaged them, so that we could give an accurate estimate about the situation of dietary BCAAs intake over a long duration. Therefore, we supposed that our findings of the nonlinear relationship between dietary BCAAs intake and increased hypertension risk were not only adequately representative, but also could fill the gap of epidemiological evidence in the field of BCAAs intake and hypertension risk.

As essential amino acids of human bodies, BCAAs play important roles in promoting protein synthesis by activating the skeletal muscle Akt/mTORC1 signal pathway [42]. The BCAAs can also promote glucose uptake via several pathways, such as by increasing the phosphorylation level of Akt and mTOR in different organs [43]. However, experimental studies have revealed that the activated mTOR was closely correlated with elevated BP. For example, angiotensin II (ANG II) can reduce the production of nitric oxide (NO) and impair insulin-induced vasodilation by activating the mTOR/S6K1 pathway [44]. Atherosclerotic lesions of vascular smooth muscle cells (VSMCs) are also associated with activated mTOR [45]. Chen et al. reported that in hypertensive rats, cardiac hypertrophy, elevated BP, as well as higher levels of mTOR were observed, while by giving an mTOR inhibitor, the BP was decreased [46]. Therefore, as we thought, BCAAs perhaps could directly result in elevated blood pressure by activating the Akt/mTORC1 signal pathway. On the other hand, it has been shown that when the plasma concentration of BCAAs becomes too high, the tissue glucose uptake could be inhibited and IR might happen [47]. Currently, a generally accepted potential mechanism underlying the relationship between BCAAs and an increased risk of chronic inflammation and IR was that there existed some toxic intermediates in the BCAAs catabolism, other than the BCAAs themselves triggering the occurrence of IR. For instance, in skeletal muscle, Val can be decomposed into an intermediate, i.e., 3-HIB, which can promote the skeletal muscle uptake of fatty acids from plasma by regulating the fatty acid transporters 3 and 4 [47]. The BCKAs produced in skeletal muscle can also be transported into the liver, where the BCKAs are thereafter decomposed into large amounts of acyl-CoA, which can produce acylcarnitine and impair the TCA cycle, resulting in oxidative stress and liver IR [5,6,7]. Accordingly, chronic inflammation and IR might also contribute to the association between BCAAs and elevated risk of CVDs, including hypertension. It has been reported that the elevated circulating level of BCAAs in a proper range might be beneficial for left ventricular function, while defective BCAAs catabolism and BCKAs accumulation could be linked to a dysfunctional heart [12,48]. By summarizing the relevant evidence above mentioned, we thought that there might be a threshold for BCAAs in triggering the harmful effects of increasing the risk of hypertension and CVD. However, the analysis of nonlinearity referring to the relationship between dietary BCAAs intake and hypertension risk has never been given in previous studies.

In our study, by performing COX regression analyses, the adverse effects on increasing hypertension risk could not be found in a relative lower range of dietary BCAAs intake in either men or women (Table 4). In further analysis using RCS models, nonlinear relationships were observed, nevertheless no significant *p*-value of nonlinearity was found in women. As we know, the nonlinear association between dietary BCAAs intake and hypertension risk has never been reported until we performed the current study. What we found verified our hypothesis that hypertension risk could increase once the dietary BCAAs intake exceeded a proper range, and gave us a new understanding about the pathogenic effects of BCAAs. We thought that our findings would be helpful for researchers and governments in developing some favorable guidance and strategies about the primary prevention of hypertension, based on dietary interventions. Meanwhile, people with daily BCAAs supplements could be well alerted that adverse effects of excessive BCAAs intake might potentially exist.

Another key point in our study was that we also performed stratified analyses to find out if the association between BCAAs and hypertension was confounded by relevant traditional risk factors. Our findings in Figure 2 elucidated that those participants with higher intakes of sodium, energy, fat, and protein showed larger HRs of hypertension risk, despite the fact that the *p*-value for interaction was only shown for dietary fat intake. High intake levels of dietary salt and sodium have been generally considered as risk factors for hypertension [49]. Consistently, in our study, those participants with a higher level of sodium intake showed a slightly higher incidence of hypertension, which perhaps contributed to the larger HR of BCAAs among these people. On the other hand, BCAAs are mainly derived from several specific foods, such as meat, poultry, egg, and dairy products, all of which are also rich in fat, as well as energy and protein [50,51,52]. As a result, higher dietary intakes of BCAAs perhaps happened in those specific participants, and thereafter increased the hypertension risk. Moreover, although similar HRs for hypertension risk were found in men and women, the associations between BP values and per SD increase in BCAAs were stronger in men, especially for the change of SBP (Table 2). Such findings could be partially attributed to the fact that the dietary intake levels of carbohydrate, fat, and protein, as well as BCAAs, were dramatically higher in our specific male participants (Table 1). 

Comparing the strengths of associations with hypertension among Ile, Leu, and Val has been performed in previous studies. For example, Mahbub et al. found that among Japanese participants, by using the categorical variable of BCAAs based on quartiles, Leu was more strongly associated with hypertension prevalence than Ile and Val [19]. In another study from Iran, only the dietary intake of Val, other than Ile or Leu, was found to be associated with hypertension [41]. In our study, by standardizing the dietary intake levels of all three BCAAs using SD, we found that associations of both BP values and hypertension risk with each individual BCAA existed with statistical significance, and no obviously stronger association was found, especially in the analysis of hypertension risk. Such different findings between previous studies and ours probably could be due to the variance in the populations with different backgrounds of genetics and dietary habits. Nevertheless, we thought that our findings could still provide some evidence for future research focusing on the specific biological functions of different BCAAs. 

Several limitations in our study should be noted. Firstly, since our study was performed among the Chinese population, such results might not be well generalized in other populations in different backgrounds. Secondly, our results only suggested that in the case of a relatively high level of dietary BCAAs intake could the adverse effects of increased hypertension risk be seen, while an accurate recommended range of dietary BCAAs intake could not be proposed, which will thus depend on further experimental studies and relevant randomized controlled clinical trials. Thirdly, our findings about the nonlinear associations were derived based on the investigation using FFQ, whose accuracy in estimating the intake level of BCAAs was not validated due to the lack of relevant examinations on blood samples. However, several strengths in our study could still be listed as follows. Firstly, as far as we know, our study was the first one to disclose the causality between BCAAs intake and hypertension risk in a large sample size, implying that our findings could be conclusive. Secondly, dietary intakes of BCAAs were estimated at different time points and then averaged, which could well reflect the effect of dietary BCAAs intake in a longer duration on hypertension risk in each individual participant. Last but not least, all three of the BCAAs were separately analyzed in our study, other than the overall level of the BCAAs, so that we could well understand the associations between different individual BCAA and hypertension risk.

## 5. Conclusions

Both elevated BP and hypertension risk were positively associated with the dietary intakes of all three of the BCAAs in general Chinese residents, and the nonlinear relationship between BCAAs and hypertension risk existed, especially in men. Our findings implied that there existed a threshold for BCAAs intake in increasing the hypertension risk, and could provide some concrete evidence in the primary prevention of hypertension based on dietary interventions.

## Figures and Tables

**Table 1:** General characteristics of participants (*n* = 8491).

	Men (*n* = 3995)	Women (*n* = 4496)	*p*-Value
Age at baseline (years old)	44.4 ± 14.2	44.8 ± 13.9	0.148
Follow-up duration (years)	4.68 ± 2.13	4.81 ± 2.16	0.005
Weight at baseline (kg)	63.4 ± 10.3	55.4 ± 8.87	<0.001
Height at baseline (cm)	166.9 ± 6.6	155.0 ± 6.3	<0.001
BMI at baseline (kg/m^2^)	22.5 ± 3.8	22.6 ± 3.7	0.168
WC at baseline (cm)	81.7 ± 9.3	78.1 ± 9.1	<0.001
Total physical activity at baseline (log-transformed METs-hours/week)	4.02 ± 1.95	3.35 ± 1.87	<0.001
Energy intake at baseline (Kcal/day)	2390.9 ± 685.6	2036.1 ± 610.3	<0.001
Carbohydrate intake at baseline (g/day)	347.9 ± 115.3	296.0 ± 100.7	<0.001
Fat intake at baseline (g/day)	74.1 ± 39.3	66.5 ± 38.0	<0.001
Protein intake at baseline (g/day)	72.5 ± 26.1	62.7 ± 23.0	<0.001
Salt intake (g/day)	9.0 ± 12.8	9.4 ± 13.8	0.217
Sodium intake (g/day)	5.9 ± 7.1	6.0 ± 7.4	0.409
Ile intake during the follow-up (g/day)	2.44 ± 1.16	2.25 ± 1.06	<0.001
Leu intake during the follow-up (g/day)	4.39 ± 2.18	4.05 ± 2.01	<0.001
Val intake during the follow-up (g/day)	2.88 ± 1.37	2.65 ± 1.26	<0.001
SBP at baseline (mmHg)	117.3 ± 10.4	113.5 ± 11.9	<0.001
DBP at baseline (mmHg)	76.3 ± 7.5	73.9 ± 8.1	<0.001
SBP at the end of follow-up (mmHg)	123.6 ± 15.4	120.5 ± 17.0	<0.001
DBP at the end of follow-up (mmHg)	80.8 ± 10.5	77.6 ± 10.5	<0.001
Change of SBP during the follow-up (mmHg)	6.3 ± 15.5	6.9 ± 16.5	0.117
Change of DBP during the follow-up (mmHg)	4.4 ± 10.8	3.7 ± 11.0	0.006
Urban residents (%)	35.1	35.4	0.839
Han ethnicity (%)	86.6	86.6	0.716
Education (%)			<0.001
Illiteracy	7.7	19.4	
Primary school	19.3	21.3	
Middle school	73	59.3	
High school or above	32.5	23.1	
Smoking (%)	63.8	3.7	<0.001
Alcohol drinking (%)	59.3	8.9	<0.001
T2DM diagnosed at baseline (%)	0.8	1	0.517
Anti-hypertensive treatment (%)	3.1	4.5	0.005
Incidence of hypertension (%)	28.9	24.6	<0.001

Values are presented as mean ± SD, or %. Abbreviations: BMI: body mass index; WC: waist circumference; MET: metabolic equivalent; Ile: isoleucine; Leu: leucine; Val: valine; SBP: systolic blood pressure; DBP: diastolic blood pressure; T2DM: type 2 diabetes mellitus.

**Table 2:** Adjusted associations between BP and per SD increase in BCAAs in participants without antihypertensive treatment (*n* = 7761).

	Men (*n* = 3646)				Women (*n* = 4115)				*p*-Value for Interaction
	Coefficient	95% CI	*p*-Value	R^2^	Coefficient	95% CI	*p*-Value	R^2^	
SBP									
Ile	2.55	1.84, 3.25	<0.001	0.102	1.82	1.16, 2.49	<0.001	0.194	0.183
Leu	2.39	1.70, 3.07	<0.001	0.100	1.80	1.15, 2.45	<0.001	0.195	0.287
Val	2.40	1.71, 3.09	<0.001	0.100	1.74	1.09, 2.39	<0.001	0.194	0.249
DBP									
Ile	1.27	0.78, 1.76	<0.001	0.041	0.97	0.52, 1.42	<0.001	0.076	0.429
Leu	1.21	0.73, 1.69	<0.001	0.040	0.96	0.52, 1.41	<0.001	0.076	0.546
Val	1.19	0.70, 1.67	<0.001	0.039	0.92	0.48, 1.37	<0.001	0.076	0.526
Change of SBP									
Ile	2.46	1.74, 3.19	<0.001	0.070	1.31	0.60, 2.02	<0.001	0.064	0.008
Leu	2.30	1.59, 3.00	<0.001	0.068	1.33	0.63, 2.02	<0.001	0.064	0.020
Val	2.38	1.67, 3.10	<0.001	0.069	1.29	0.59, 1.98	<0.001	0.064	0.012
Change of DBP									
Ile	1.16	0.64, 1.69	<0.001	0.045	0.76	0.27, 1.26	0.003	0.038	0.344
Leu	1.10	0.59, 1.61	<0.001	0.044	0.82	0.33, 1.30	0.001	0.039	0.495
Val	1.20	0.69, 1.71	<0.001	0.046	0.83	0.35, 1.32	0.001	0.039	0.399

Regression models were adjusted for age, ethnicity, education, urban residents, diagnosed T2DM, physical activity, smoking, alcohol drinking, energy, carbohydrate, fat, protein, and salt intake. The interaction by gender was only estimated for BCAAs, not for any other confounder. Abbreviations: BP: blood pressure; SD: standard deviation; BCAA: branched-chain amino acid; 95% CI: 95% confidence interval; R^2^: coefficient of determination; SBP: systolic blood pressure; DBP: diastolic blood pressure; Ile: isoleucine; Leu: leucine; Val: valine.

**Table 3:** Adjusted associations between hypertension incidence and per SD increase in BCAAs (*n* = 8491).

	Men (*n* = 3995)			Women (*n* = 4496)			*p*-Value for Interaction
	HR	95% CI	*p*-Value	HR	95% CI	*p*-Value	
Model 1							
Ile	1.23	1.16, 1.29	<0.001	1.23	1.17, 1.29	<0.001	0.803
Leu	1.22	1.16, 1.28	<0.001	1.18	1.13, 1.24	<0.001	0.700
Val	1.22	1.16, 1.28	<0.001	1.22	1.16, 1.28	<0.001	0.806
Model 2							
Ile	1.18	1.10, 1.27	<0.001	1.22	1.14, 1.31	<0.001	0.449
Leu	1.18	1.10, 1.26	<0.001	1.21	1.13, 1.30	<0.001	0.397
Val	1.18	1.10, 1.27	<0.001	1.22	1.13, 1.30	<0.001	0.376
Model 3							
Ile	1.24	1.14, 1.35	<0.001	1.29	1.19, 1.40	<0.001	0.328
Leu	1.23	1.13, 1.33	<0.001	1.27	1.18, 1.38	<0.001	0.283
Val	1.23	1.13, 1.33	<0.001	1.28	1.18, 1.38	<0.001	0.265

Ile, Leu, and Val were analyzed in separate regression models. Model 1: adjusted for age; Model 2: adjusted for ethnicity, education, urban residents, diagnosed T2DM, physical activity, smoking and alcohol drinking in addition to Model 1; Model 3: adjusted for intakes of energy, carbohydrate, fat, and salt intake in addition to Model 2. The interaction by gender was only estimated for BCAAs, not for any other confounder. Abbreviations: SD: standard deviation; BCAA: branched-chain amino acid; HR: hazard ratio; 95% CI: 95% confidence interval; Ile: isoleucine; Leu: leucine; Val: valine.

**Table 4:** Adjusted associations between hypertension incidence and continuous variables of BCAAs in subgroups divided using quartiles (*n* = 8491).

Men (*n* = 3995)						Women (*n* = 4496)						*p*-Value for Interaction
	*n*	Cases (Incidence)	HR	95% CI	*p*-Value		*n*	Cases (Incidence)	HR	95% CI	*p*-Value	
Ile (g/day)						Ile (g/day)						
Q1 (<1.66)	1001	272 (27.2)	0.85	0.48, 1.51	0.575	Q1 (<1.57)	1132	257 (22.7)	1.21	0.63, 2.31	0.567	0.709
Q2 (1.66–2.32)	994	230 (23.1)	1.28	0.42, 3.90	0.665	Q2 (1.57–2.15)	1119	246 (22.0)	1.99	0.72, 5.52	0.187	0.384
Q3 (2.32–3.06)	997	267 (26.8)	1.60	0.70, 3.67	0.265	Q3 (2.15–2.87)	1128	255 (22.6)	1.84	0.80, 4.25	0.154	0.933
Q4 (≥3.06)	1003	386 (38.5)	1.14	1.01, 1.29	0.041	Q4 (≥2.87)	1117	346 (31.0)	1.36	1.20, 1.54	<0.001	0.117
Leu (g/day)						Leu (g/day)						
Q1 (<2.94)	998	272 (27.3)	0.94	0.66, 1.33	0.939	Q1 (<2.76)	1118	259 (23.2)	1.17	0.81, 1.70	0.400	0.503
Q2 (2.94–4.12)	1004	238 (23.7)	0.84	0.48, 1.47	0.835	Q2 (2.76–3.82)	1127	241 (21.4)	2.06	1.10, 3.85	0.024	0.020
Q3 (4.12–5.54)	997	264 (26.5)	1.16	0.74, 1.81	0.530	Q3 (3.82–5.15)	1133	255 (22.5)	1.05	0.67, 1.64	0.839	0.950
Q4 (≥5.54)	996	381 (38.3)	1.09	1.03, 1.15	0.003	Q4 (≥5.15)	1118	349 (31.2)	1.15	1.08, 1.23	<0.001	0.191
Val (g/day)						Val (g/day)						
Q1 (<1.96)	999	263 (26.3)	0.93	0.56, 1.54	0.928	Q1 (<1.85)	1122	250 (22.3)	1.27	0.71, 2.26	0.419	0.745
Q2 (1.96–2.71)	1000	237 (23.7)	0.63	0.26, 1.53	0.305	Q2 (1.85–2.52)	1123	236 (21.0)	1.86	0.72, 4.85	0.203	0.149
Q3 (2.71–3.61)	991	282 (28.5)	2.49	1.30, 4.76	0.006	Q3 (2.52–3.37)	1126	267 (23.7)	0.86	0.42, 1.76	0.671	0.023
Q4 (≥3.61)	1005	373 (37.1)	1.15	1.03, 1.27	0.009	Q4 (≥3.37)	1125	351 (31.2)	1.25	1.13, 1.39	<0.001	0.475

Ile, Leu, and Val were analyzed in separate regression models. Regression models were adjusted for age, ethnicity, education, urban residents, diagnosed T2DM, physical activity, smoking, alcohol drinking, energy, carbohydrate, fat, protein, and salt intake. The interaction by gender was only estimated for BCAAs, not for any other confounder. Abbreviations: SD: standard deviation; BCAA: branched-chain amino acid; HR: hazard ratio; 95% CI: 95% confidence interval; Ile: isoleucine; Leu: leucine; Val: valine.

## Data Availability

The datasets analyzed for this study can be found in China Health and Nutrition Survey (https://www.cpc.unc.edu/projects/china, accessed on 9 October 2019).

## Supplementary Materials

The following supporting information can be downloaded at: https://www.mdpi.com/article/10.3390/nu14132582/s1, Figure S1: Flow chart of the prospective longitudinal analysis; Figure S2: Kaplan–Meier curve of the probability of non-hypertension in men and women; Figure S3: Diagnostic of proportional hazards assumption based on weighted Schoenfeld residuals. The beta (time) was the time-varying coefficient calculated based on weighted Schoenfeld residuals and regression coefficient of COX regression models for each individual BCAA. The Pearson correlation was performed to estimate the relationship between residual and time rank. Correlation coefficients and relevant *p*-values were given; Table S1: Adjusted associations between BP and categorical variables of BCAAs based on quartiles in participants without antihypertensive treatment (*n* = 7761); Table S2: Adjusted associations between BP and categorical variables of BCAAs based on quartiles in participants without antihypertensive treatment (*n* = 7761); Table S3: Probability of non-hypertension estimated using Kaplan–Meier method (*n* = 8491); Table S4: Adjusted associations between hypertension incidence and categorical variables of BCAAs based on quartiles (*n* = 8491).
