# Supplementary material for: Association between Excessive Dietary Branched-Chain Amino Acids Intake and Hypertension Risk in Chinese Population
Source: Nutrients. 2022 Jun 22;14(13):2582. doi: 10.3390/nu14132582 (PMC9268479; doi:10.3390/nu14132582)
Supplement: Supplementary file 1 [file nutrients-14-02582-s001.zip › nutrients-1759848-supplementary.pdf]

Association between excessive dietary branched-chain amino acids intake and hypertension risk in Chinese population

No. of tables: 4 tables and 3 figures

Table S1. Adjusted associations of BP with categorical variables of BCAAs based on quartiles in participants without antihypertensive treatment (n=7,761)

	Men (n=3,646)							Women (n=4,115)						*P*-value for interaction
	n	mean ± SD	Coefficient	95% CI	*P*-value	R^2		n	mean ± SD	Coefficient	95% CI	*P*-value	R^2	
SBP														
Ile (g/day)						0.106	Ile (g/day)						0.191	0.004
Q1 (<1.66)	905	121.2±15.2		Ref			Q1 (<1.57)	1,027	118.6±16.9		Ref			
Q2 (1.66-2.32)	903	122.0±14.5	1.33	-0.51, 3.16	0.157		Q2 (1.57-2.15)	1,036	119.0±16.6	1.15	-0.58, 2.87	0.193		
Q3 (2.32-3.06)	925	123.0±14.3	3.04	1.18, 4.89	0.001		Q3 (2.15-2.87)	1,036	118.9±14.9	0.99	-0.81, 2.79	0.281		
Q4 (≥3.06)	913	126.1±15.3	7.16	5.18, 9.13	<0.001		Q4 (≥2.87)	1,016	121.7±16.6	4.11	2.18, 6.04	<0.001		
Leu (g/day)						0.107	Leu (g/day)						0.190	0.004
Q1 (<2.94)	900	121.3±15.3		Ref			Q1 (<2.76)	1,014	118.5±17.0		Ref			
Q2 (2.94-4.12)	916	121.7±14.2	0.90	-0.97, 2.76	0.346		Q2 (2.76-3.82)	1,045	119.1±16.5	1.57	-0.18, 3.32	0.078		
Q3 (4.12-5.54)	919	122.9±14.2	2.97	1.09, 4.85	0.002		Q3 (3.82-5.15)	1,042	119.0±15.0	1.51	-0.29, 3.30	0.100		
Q4 (≥5.54)	911	126.3±15.4	7.01	5.02, 9.00	<0.001		Q4 (≥5.15)	1,014	121.6±16.5	3.98	2.08, 5.89	<0.001		
Val (g/day)						0.102	Val (g/day)						0.191	0.017
Q1 (<1.96)	907	121.3±15.0		Ref			Q1 (<1.85)	1,020	118.4±16.7		Ref			
Q2 (1.96-2.71)	905	121.9±14.3	0.85	-0.96, 2.65	0.357		Q2 (1.85-2.52)	1,037	119.1±16.5	1.70	-0.01, 3.40	0.051		
Q3 (2.71-3.61)	918	123.1±14.5	3.29	1.43, 5.16	0.001		Q3 (2.52-3.37)	1,036	119.1±15.3	1.41	-0.38, 3.20	0.122		
Q4 (≥3.61)	916	126.0±15.4	6.42	4.45, 8.39	<0.001		Q4 (≥3.37)	1,022	121.6±16.6	4.27	2.36, 6.18	<0.001		
DBP														
Ile (g/day)						0.042	Ile (g/day)						0.072	0.041
Q1 (<1.66)	905	79.4±10.6		Ref			Q1 (<1.57)	1,027	76.4±10.7		Ref			
Q2 (1.66-2.32)	903	79.2±9.5	-0.26	-1.55, 1.03	0.689		Q2 (1.57-2.15)	1,036	76.8±10.0	0.83	-0.35, 2.01	0.166		
Q3 (2.32-3.06)	925	80.8±10.0	0.80	-0.51, 2.10	0.230		Q3 (2.15-2.87)	1,036	76.8±10.0	0.36	-0.86, 1.59	0.563		
Q4 (≥3.06)	913	82.5±9.9	2.96	1.57, 4.35	<0.001		Q4 (≥2.87)	1,016	78.4±9.8	1.77	0.45, 3.09	0.009		
Leu (g/day)						0.040	Leu (g/day)						0.071	0.042

Q1 (<2.94)	900	79.4±10.6		Ref			Q1 (<2.76)	1,014	76.5±10.7		Ref			
Q2 (2.94-4.12)	916	79.3±9.6	-0.34	-1.65, 0.97	0.607		Q2 (2.76-3.82)	1,045	76.7±10.0	0.87	-0.32, 2.07	0.152		
Q3 (4.12-5.54)	919	80.7±9.9	0.48	-0.84, 1.80	0.474		Q3 (3.82-5.15)	1,042	77.0±9.9	0.53	-0.70, 1.75	0.399		
Q4 (≥5.54)	911	82.5±10.0	2.65	1.25, 4.05	<0.001		Q4 (≥5.15)	1,014	78.4±9.9	1.39	0.09, 2.69	0.036		
Val (g/day)						0.038	Val (g/day)						0.073	0.126
Q1 (<1.96)	907	79.3±10.5		Ref			Q1 (<1.85)	1,020	76.4±10.6		Ref			
Q2 (1.96-2.71)	905	79.5±9.4	0.09	-1.18, 1.35	0.893		Q2 (1.85-2.52)	1,037	76.8±10.1	0.99	-0.17, 2.16	0.094		
Q3 (2.71-3.61)	918	80.8±10.2	1.19	-0.12, 2.50	0.075		Q3 (2.52-3.37)	1,036	77.0±9.9	0.62	-0.61, 1.84	0.323		
Q4 (≥3.61)	916	82.3±10.0	2.67	1.29, 4.06	<0.001		Q4 (≥3.37)	1,022	78.4±10.0	1.94	0.63, 3.24	0.004		

Regression models were adjusted for age, ethnicity, education, urban residents, diagnosed T2DM, physical activity, smoking, alcohol drinking, energy, carbohydrate, fat, protein, and salt intake. The interaction by gender was only estimated for BCAAs, not for any other confounder. **Abbreviations:** BP: blood pressure; BCAA: branched-chain amino acid; SD: standard deviation; 95% CI: 95% confidence interval; R^2: coefficient of determination; SBP: systolic blood pressure; DBP: diastolic blood pressure; Ile: isoleucine; Leu: leucine; Val: valine.

Table S2. Adjusted associations of BP with categorical variables of BCAAs based on quartiles in participants without antihypertensive treatment (n=7,761)

	Men (n=3,646)							Women (n=4,115)						*P*-value for interaction
	n	mean ± SD	Coefficient	95% CI	*P*-value	R^2		n	mean ± SD	Coefficient	95% CI	*P*-value	R^2	
Change of SBP														
Ile (g/day)						0.071	Ile (g/day)						0.064	<0.001
Q1 (<1.66)	905	4.36±15.4		Ref			Q1 (<1.57)	1,027	6.50±16.36		Ref			
Q2 (1.66-2.32)	903	5.33±14.9	1.24	-0.68, 3.15	0.207		Q2 (1.57-2.15)	1,036	5.65±16.10	-0.41	-2.25, 1.42	0.659		
Q3 (2.32-3.06)	925	5.90±14.6	3.01	1.07, 4.95	0.002		Q3 (2.15-2.87)	1,036	5.84±15.86	-0.70	-2.62, 1.22	0.477		
Q4 (≥3.06)	913	8.03±15.4	6.61	4.55, 8.67	<0.001		Q4 (≥2.87)	1,016	7.51±15.91	2.44	0.38, 4.50	0.020		
Leu (g/day)						0.072	Leu (g/day)						0.062	<0.001
Q1 (<2.94)	900	4.46±15.33		Ref			Q1 (<2.76)	1,014	6.48±16.28		Ref			
Q2 (2.94-4.12)	916	5.29±15.06	1.75	-0.18, 3.69	0.076		Q2 (2.76-3.82)	1,045	6.01±16.17	0.15	-1.72, 2.01	0.876		
Q3 (4.12-5.54)	919	5.49±14.29	2.90	0.94, 4.86	0.004		Q3 (3.82-5.15)	1,042	5.50±15.93	-0.27	-2.19, 1.65	0.784		
Q4 (≥5.54)	911	8.39±15.45	6.89	4.82, 8.95	<0.001		Q4 (≥5.15)	1,014	7.53±15.85	2.33	0.30, 4.36	0.024		
Val (g/day)						0.067	Val (g/day)						0.063	0.001
Q1 (<1.96)	907	4.25±15.28		Ref			Q1 (<1.85)	1,020	6.32±16.11		Ref			
Q2 (1.96-2.71)	905	5.31±14.69	1.16	-0.72, 3.03	0.227		Q2 (1.85-2.52)	1,037	5.74±16.15	0.05	-1.77, 1.86	0.959		
Q3 (2.71-3.61)	918	5.99±14.57	3.26	1.31, 5.21	0.001		Q3 (2.52-3.37)	1,036	5.94±15.96	0.12	-1.80, 2.03	0.906		
Q4 (≥3.61)	916	8.08±15.61	5.98	3.93, 8.02	<0.001		Q4 (≥3.37)	1,022	7.49±16.02	2.70	0.66, 4.73	0.009		
Change of DBP														
Ile (g/day)						0.044	Ile (g/day)						0.036	0.063
Q1 (<1.66)	905	3.40±10.94		Ref			Q1 (<1.57)	1,027	3.21±11.24		Ref			
Q2 (1.66-2.32)	903	3.33±10.22	-0.14	-1.53, 1.24	0.842		Q2 (1.57-2.15)	1,036	2.83±10.96	-0.05	-1.34, 1.24	0.947		
Q3 (2.32-3.06)	925	4.40±10.43	1.14	-0.27, 2.54	0.112		Q3 (2.15-2.87)	1,036	3.26±10.81	-0.16	-1.51, 1.19	0.814		
Q4 (≥3.06)	913	5.39±10.27	2.62	1.14, 4.11	0.001		Q4 (≥2.87)	1,016	4.28±10.07	1.13	-0.32, 2.57	0.126		
Leu (g/day)						0.044	Leu (g/day)						0.036	0.061

Q1 (<2.94)	900	3.44±10.93		Ref			Q1 (<2.76)	1,014	3.25±11.23		Ref			
Q2 (2.94-4.12)	916	3.44±10.26	0.10	-1.30, 1.50	0.892		Q2 (2.76-3.82)	1,045	2.94±11.13	0.08	-1.22, 1.39	0.901		
Q3 (4.12-5.54)	919	4.14±10.27	0.78	-0.64, 2.20	0.280		Q3 (3.82-5.15)	1,042	3.09±10.59	-0.24	-1.58, 1.11	0.728		
Q4 (≥5.54)	911	5.52±10.40	2.63	1.14, 4.13	0.001		Q4 (≥5.15)	1,014	4.32±10.13	1.03	-0.39, 2.45	0.154		
Val (g/day)						0.043	Val (g/day)						0.037	0.167
Q1 (<1.96)	907	3.17±10.81		Ref			Q1 (<1.85)	1,020	3.09±11.09		Ref			
Q2 (1.96-2.71)	905	3.62±10.06	0.55	-0.80, 1.91	0.424		Q2 (1.85-2.52)	1,037	2.83±11.03	0.15	-1.12, 1.42	0.818		
Q3 (2.71-3.61)	918	4.38±10.63	1.68	0.27, 3.08	0.019		Q3 (2.52-3.37)	1,036	3.28±10.76	0.29	-1.05, 1.63	0.674		
Q4 (≥3.61)	916	5.36±10.36	2.67	1.19, 4.15	<0.001		Q4 (≥3.37)	1,022	4.39±10.19	1.60	0.18, 3.03	0.028		

Regression models were adjusted for age, ethnicity, education, urban residents, diagnosed T2DM, physical activity, smoking, alcohol drinking, energy, carbohydrate, fat, protein, and salt intake. The interaction by gender was only estimated for BCAAs, not for any other confounder. **Abbreviations:** BP: blood pressure; BCAA: branched-chain amino acid; SD: standard deviation; 95% CI: 95% confidence interval; R^2: coefficient of determination; SBP: systolic blood pressure; DBP: diastolic blood pressure; Ile: isoleucine; Leu: leucine; Val: valine.

Table S3. Probability of non-hypertension estimated using Kaplan-Meier method (n=8,491)

Time (years)	No. of participants at risk	No. of participants diagnosed as hypertension	Probability of non-hypertension	SE	95%CI
		Men			
2	3,995	454	0.886	0.005	0.877, 0.896
3	2,690	78	0.861	0.006	0.850, 0.872
5	2,505	434	0.712	0.008	0.696, 0.727
7	1,494	189	0.622	0.009	0.604, 0.640
		Women			
2	4,496	426	0.905	0.004	0.897, 0.914
3	3,069	73	0.884	0.005	0.874, 0.893
5	2,889	410	0.758	0.007	0.744, 0.772
7	1,884	195	0.68	0.008	0.664, 0.696

Abbreviations: SE: standard error; 95%CI: 95% confidence interval.

Table S4. Adjusted associations of hypertension incidence with categorical variables of BCAAs based on quartiles (n=8,491)

	Men (n=3,995)						Women (n=4,496)					*P*-value for interaction
	n	Cases (incidence)	HR	95% CI	*P*-value		n	Cases (incidence)	HR	95% CI	*P*-value	
Model 1												
Ile (g/day)						Ile (g/day)						0.416
Q1 (<1.66)	1,001	272 (27.2)		Ref		Q1 (<1.57)	1,132	257 (22.7)		Ref		
Q2 (1.66-2.32)	994	230 (23.1)	0.84	0.71, 1.01	0.059	Q2 (1.57-2.15)	1,119	246 (22.0)	1.00	0.84, 1.19	0.970	
Q3 (2.32-3.06)	997	267 (26.8)	0.98	0.83, 1.16	0.788	Q3 (2.15-2.87)	1,128	255 (22.6)	1.02	0.86, 1.21	0.829	
Q4 (≥3.06)	1,003	386 (38.5)	1.53	1.31, 1.79	<0.001	Q4 (≥2.87)	1,117	346 (31.0)	1.54	1.31, 1.81	<0.001	
Leu (g/day)						Leu (g/day)						0.566
Q1 (<2.94)	998	272 (27.3)		Ref		Q1 (<2.76)	1,118	259 (23.2)		Ref		
Q2 (2.94-4.12)	1,004	238 (23.7)	0.87	0.73, 1.03	0.102	Q2 (2.76-3.82)	1,127	241 (21.4)	0.92	0.78, 1.10	0.377	
Q3 (4.12-5.54)	997	264 (26.5)	0.95	0.80, 1.13	0.547	Q3 (3.82-5.15)	1,133	255 (22.5)	0.99	0.83, 1.18	0.890	
Q4 (≥5.54)	996	381 (38.3)	1.52	1.30, 1.78	<0.001	Q4 (≥5.15)	1,118	349 (31.2)	1.51	1.29, 1.78	<0.001	
Val (g/day)						Val (g/day)						0.803
Q1 (<1.96)	999	263 (26.3)		Ref		Q1 (<1.85)	1,122	250 (22.3)		Ref		
Q2 (1.96-2.71)	1,000	237 (23.7)	0.89	0.74, 1.06	0.177	Q2 (1.85-2.52)	1,123	236 (21.0)	0.97	0.81, 1.15	0.700	
Q3 (2.71-3.61)	991	282 (28.5)	1.06	0.89, 1.25	0.537	Q3 (2.52-3.37)	1,126	267 (23.7)	1.06	0.89, 1.26	0.536	
Q4 (≥3.61)	1,005	373 (37.1)	1.49	1.27, 1.74	<0.001	Q4 (≥3.37)	1,125	351 (31.2)	1.55	1.32, 1.83	<0.001	
Model 2												
Ile (g/day)						Ile (g/day)						0.636
Q1 (<1.66)	1,001	272 (27.2)		Ref		Q1 (<1.57)	1,132	257 (22.7)		Ref		
Q2 (1.66-2.32)	994	230 (23.1)	0.76	0.59, 0.98	0.031	Q2 (1.57-2.15)	1,119	246 (22.0)	1.12	0.88, 1.43	0.356	
Q3 (2.32-3.06)	997	267 (26.8)	0.77	0.61, 0.99	0.039	Q3 (2.15-2.87)	1,128	255 (22.6)	1.00	0.78, 1.28	0.984	
Q4 (≥3.06)	1,003	386 (38.5)	1.35	1.09, 1.67	0.006	Q4 (≥2.87)	1,117	346 (31.0)	1.48	1.18, 1.86	0.001	
Leu (g/day)						Leu (g/day)						0.726

Q1 (<2.94)	998	272 (27.3)	Ref			Q1 (<2.76)	1,118	259 (23.2)	Ref			
Q2 (2.94-4.12)	1,004	238 (23.7)	0.78	0.60, 1.01	0.055	Q2 (2.76-3.82)	1,127	241 (21.4)	0.98	0.76, 1.26	0.858	
Q3 (4.12-5.54)	997	264 (26.5)	0.76	0.59, 0.98	0.031	Q3 (3.82-5.15)	1,133	255 (22.5)	0.98	0.77, 1.26	0.879	
Q4 (≥5.54)	996	381 (38.3)	1.31	1.05, 1.64	0.016	Q4 (≥5.15)	1,118	349 (31.2)	1.41	1.12, 1.77	0.003	
Val (g/day)						Val (g/day)						0.918
Q1 (<1.96)	999	263 (26.3)	Ref			Q1 (<1.85)	1,122	250 (22.3)	Ref			
Q2 (1.96-2.71)	1,000	237 (23.7)	0.84	0.65, 1.07	0.162	Q2 (1.85-2.52)	1,123	236 (21.0)	1.03	0.80, 1.32	0.826	
Q3 (2.71-3.61)	991	282 (28.5)	0.91	0.72, 1.16	0.459	Q3 (2.52-3.37)	1,126	267 (23.7)	1.08	0.84, 1.38	0.563	
Q4 (≥3.61)	1,005	373 (37.1)	1.35	1.08, 1.68	0.008	Q4 (≥3.37)	1,125	351 (31.2)	1.51	1.20, 1.90	<0.001	
Model 3												
Ile (g/day)						Ile (g/day)						0.788
Q1 (<1.66)	1,001	272 (27.2)	Ref			Q1 (<1.57)	1,132	257 (22.7)	Ref			
Q2 (1.66-2.32)	994	230 (23.1)	0.80	0.61, 1.03	0.085	Q2 (1.57-2.15)	1,119	246 (22.0)	1.18	0.92, 1.52	0.195	
Q3 (2.32-3.06)	997	267 (26.8)	0.83	0.64, 1.07	0.141	Q3 (2.15-2.87)	1,128	255 (22.6)	1.08	0.83, 1.41	0.556	
Q4 (≥3.06)	1,003	386 (38.5)	1.54	1.20, 1.97	0.001	Q4 (≥2.87)	1,117	346 (31.0)	1.66	1.27, 2.16	<0.001	
Leu (g/day)						Leu (g/day)						0.873
Q1 (<2.94)	998	272 (27.3)	Ref			Q1 (<2.76)	1,118	259 (23.2)	Ref			
Q2 (2.94-4.12)	1,004	238 (23.7)	0.82	0.63, 1.07	0.143	Q2 (2.76-3.82)	1,127	241 (21.4)	1.04	0.80, 1.34	0.790	
Q3 (4.12-5.54)	997	264 (26.5)	0.82	0.63, 1.06	0.125	Q3 (3.82-5.15)	1,133	255 (22.5)	1.05	0.81, 1.37	0.706	
Q4 (≥5.54)	996	381 (38.3)	1.47	1.14, 1.89	0.003	Q4 (≥5.15)	1,118	349 (31.2)	1.56	1.20, 2.02	0.001	
Val (g/day)						Val (g/day)						0.750
Q1 (<1.96)	999	263 (26.3)	Ref			Q1 (<1.85)	1,122	250 (22.3)	Ref			
Q2 (1.96-2.71)	1,000	237 (23.7)	0.88	0.68, 1.13	0.320	Q2 (1.85-2.52)	1,123	236 (21.0)	1.08	0.84, 1.40	0.548	
Q3 (2.71-3.61)	991	282 (28.5)	0.97	0.75, 1.25	0.802	Q3 (2.52-3.37)	1,126	267 (23.7)	1.17	0.90, 1.52	0.235	
Q4 (≥3.61)	1,005	373 (37.1)	1.50	1.17, 1.92	0.001	Q4 (≥3.37)	1,125	351 (31.2)	1.69	1.30, 2.20	<0.001	

Ile, Leu and Val were analyzed in separate regression models. Model 1: adjusted for age; Model 2: adjusted for ethnicity, education, urban residents, diagnosed T2DM, physical activity, smoking and alcohol drinking in addition to Model 1; Model 3: adjusted for energy, carbohydrate, fat, protein, and salt intake in addition to Model 2. The interaction by gender was only estimated for BCAAs, not for any other confounder. **Abbreviations:** BCAA: branched-chain amino acid; HR: hazard ratio; 95% CI: 95% confidence interval; Ile: isoleucine; Leu: leucine; Val: valine.

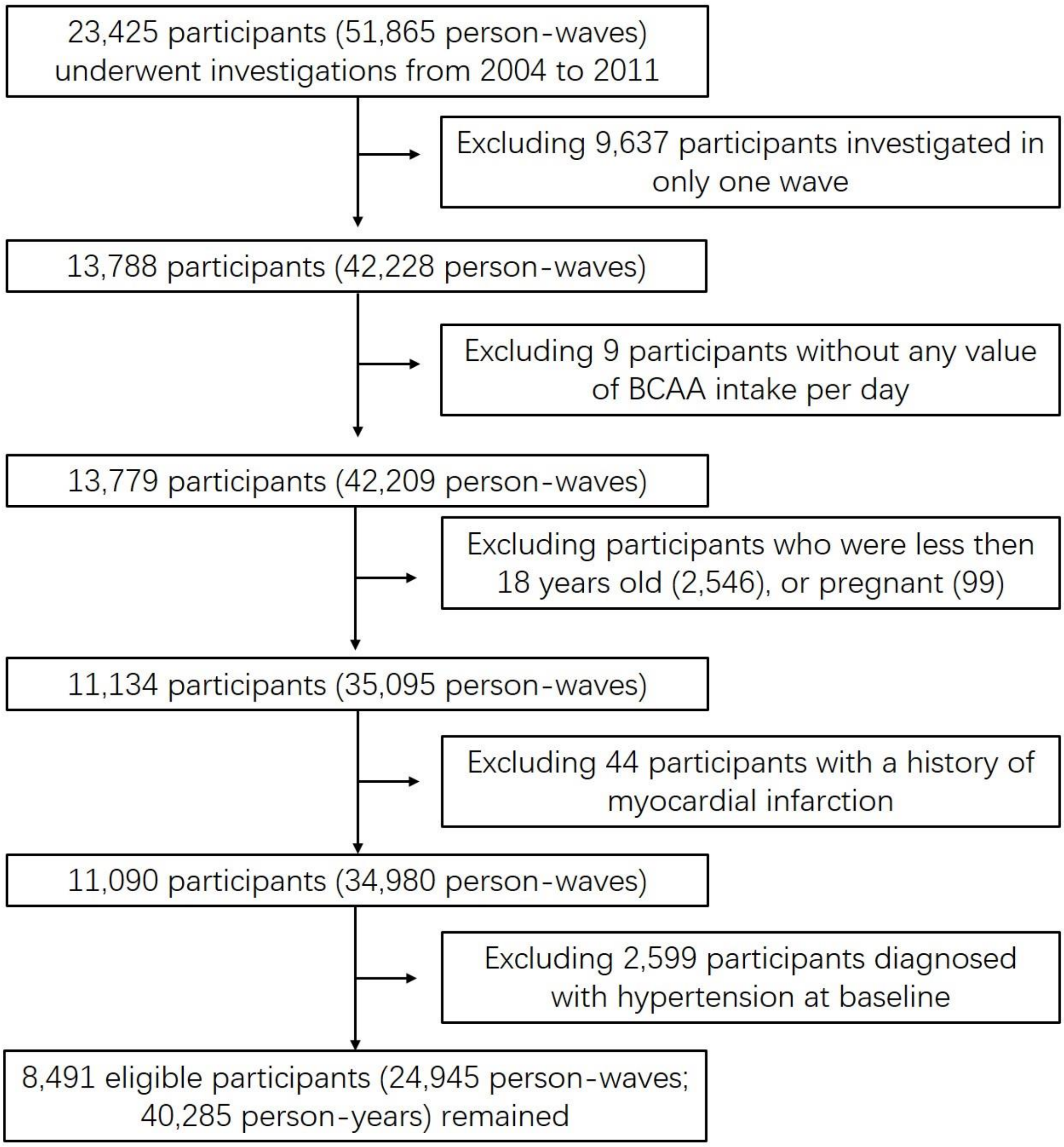

Figure S1. Flow chart of the prospective longitudinal analysis

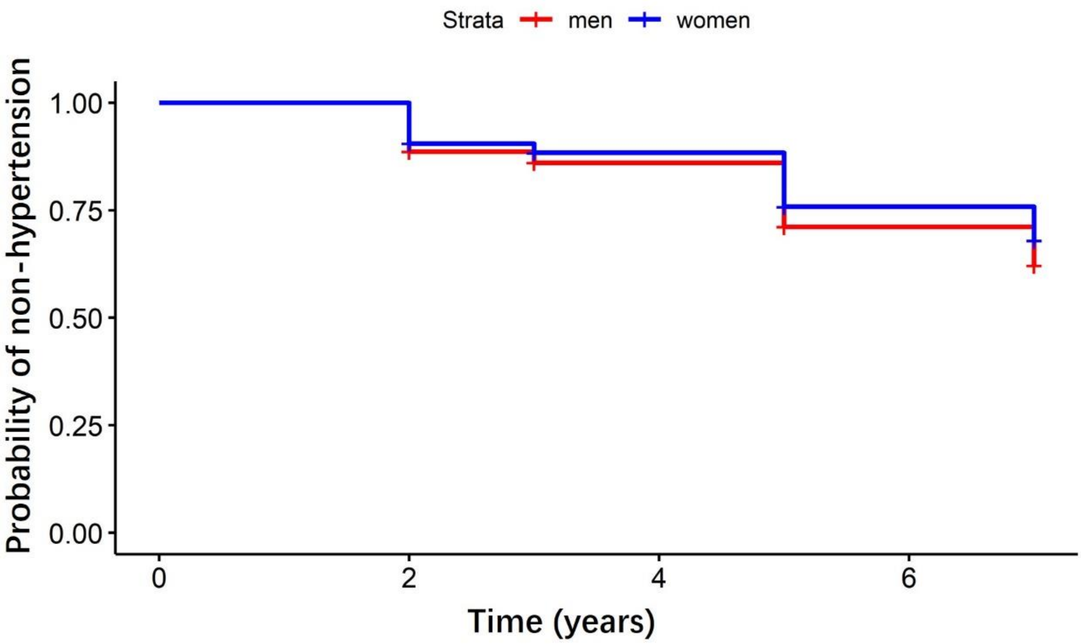

Figure S2. Kaplan-Meier curve of the probability of non-hypertension in men and women

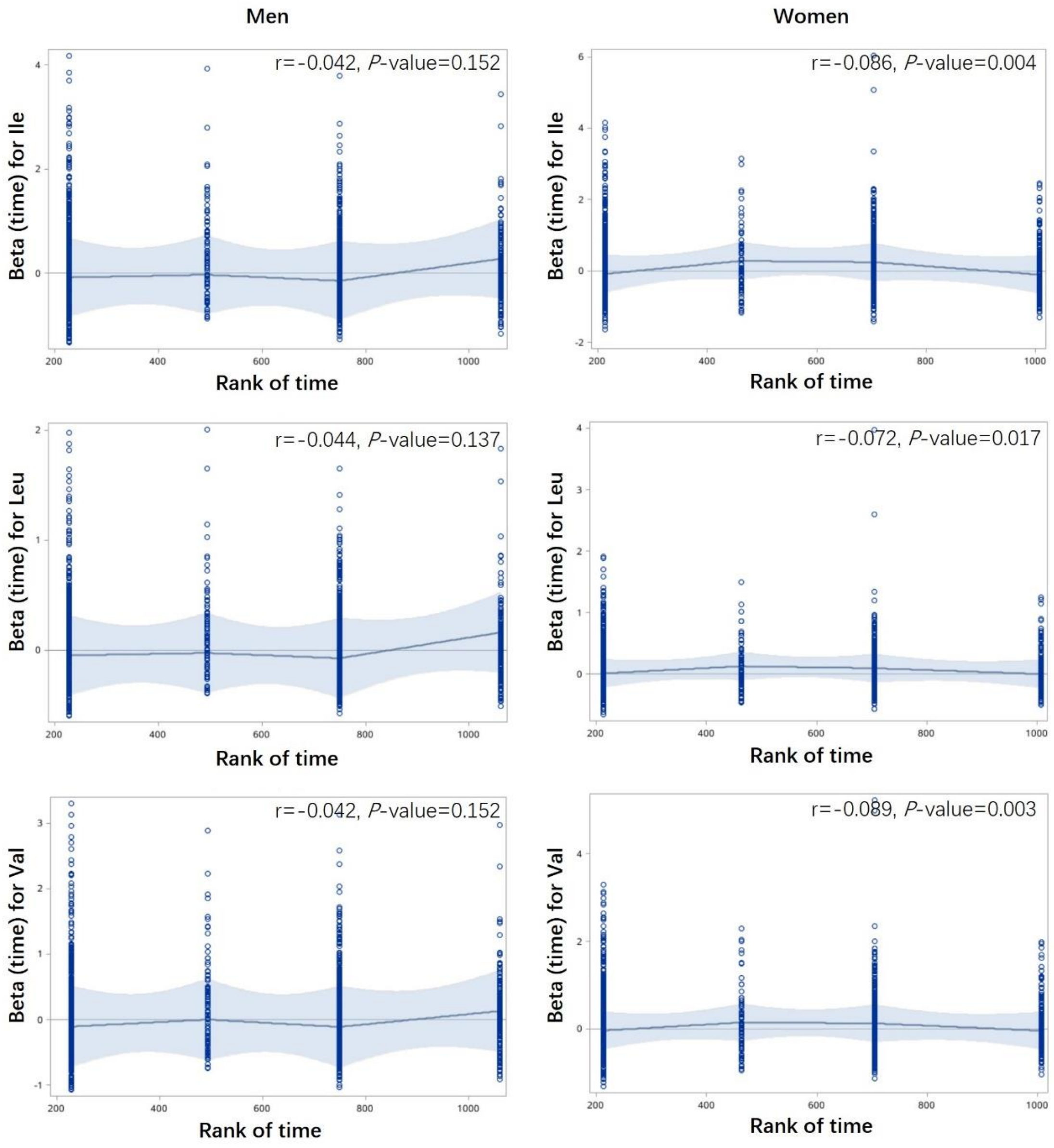

Figure S3. Diagnostic of proportional hazards assumption based on weighted Schoenfeld Residual. The beta (time) was the time-varying coefficient calculated based on weighted Schoenfeld residual and regression coefficient of COX regression models for each individual BCAA. The Pearson correlation was performed to estimate the relationship between residual and time rank. Correlation coefficients and relevant *P*-values were given.
